# Clonal evolution in gastrointestinal cancers: multi-omics insights into tumor heterogeneity, microenvironmental selection, and translational biomarkers

**DOI:** 10.3389/fonc.2026.1907210

**Published:** 2026-08-28

**Authors:** Wenyi Fan, Chunhong Zheng

**Affiliations:** 1 Frontiers Science Center for Cancer Integrative Omics, Peking University International Cancer Institute, Peking University, Beijing, China; 2 State Key Laboratory of Molecular Oncology, Beijing Key Laboratory of Cell & Gene Therapy for Solid Tumor, Peking University Cancer Hospital & Institute, Beijing, China

**Keywords:** clonal evolution, esophageal squamous cell carcinoma, gastric cancer, hepatocellular carcinoma, liquid biopsy, multi-omics, single-cell DNA sequencing, tumor microenvironment

## Abstract

Clonal evolution in hepatocellular carcinoma (HCC), esophageal squamous cell carcinoma (ESCC), and gastric cancer (GC) reflects the interaction of genetic diversification, cell-state plasticity, and tissue-specific selection. Multi-region and single-cell DNA sequencing resolve truncal and subclonal lineages, whereas single-cell and spatial transcriptomics, proteomics, and serial liquid biopsy characterize cellular states, ecological niches, and temporal dynamics. The three cancers differ in dissemination timing, dominant selective pressures, and biomarker maturity: early seeding is best supported in selected HCC cohorts, ESCC is strongly influenced by field cancerization and epithelial-stromal crosstalk, and GC follows subtype- and ecotype-dependent trajectories. We discuss the assumptions and sampling biases that constrain phylogenetic inference, the causal limits of cross-sectional tumor atlases, clonal hematopoiesis, and the incremental value of broad multi-omics over focused assays. Liquid-biopsy detection of minimal residual disease is prognostic, but treatment benefit from marker-guided intervention remains context dependent. Near-term translation requires standardized, decision-linked assays; adaptive therapy and evolutionary steering remain investigational.

## 1 Introduction

Hepatocellular carcinoma (HCC), esophageal squamous cell carcinoma (ESCC), and gastric cancer (GC) account for a substantial cancer burden, particularly in East Asia (1–4). Despite improvements in prevention, endoscopic detection, surgery, radiotherapy, systemic therapy, and molecular stratification, relapse after curative-intent treatment and progression after an initial response remain common in invasive or advanced disease (5, 6). Stage, histology, and individual driver alterations do not fully account for these outcomes.

An evolutionary framework considers how heritable and quasi-heritable variation arises, how lineages compete within a tissue-specific ecosystem, and how treatment alters that competition (7–11). Genetic alterations provide lineage information, whereas epigenetic programs, transcriptional states, metabolism, immune pressure, stromal support, and access to nutrients or vasculature influence whether a lineage expands, disseminates, remains dormant, or survives therapy. The fitness effect of a given alteration may therefore differ in a cirrhotic liver, an injured squamous field, and an immune-stromal gastric ecotype.

This review first defines truncal and subclonal architecture, neutral-like diversification, adaptive selection, bottlenecks, clonal fitness, and historical contingency, and explains how these features are inferred. It then assesses the contribution of multi-region and single-cell DNA sequencing relative to transcriptomic and spatial atlases, compares dissemination patterns and selective pressures across HCC, ESCC, and GC, and evaluates the readiness of evolution-informed biomarkers for clinical testing. Throughout, we distinguish association from causation and emphasize that clinical translation depends on prospective, action-linked validation rather than molecular complexity alone.

## 2 Conceptual and methodological foundations

### 2.1 Core terms and how they are inferred

A truncal alteration is assigned to the common ancestral lineage of the sampled cancer population. In bulk data, this assignment integrates variant allele fraction with tumor purity, local copy number, ploidy, and concordance across regions to estimate the cancer-cell fraction. A subclonal alteration is confined to a fraction of cancer cells or to one or more sample regions. A branch comprises alterations shared by a descendant lineage but absent from other sampled lineages. Single-cell DNA sequencing can assess co-occurrence within individual cells, whereas reconstruction from bulk data remains model dependent (9, 12–22).

A bottleneck is a reduction in effective population size or clonal diversity, as may occur during metastatic seeding, surgery, chemotherapy, radiotherapy, targeted therapy, or immune selection. A selection pressure is an environmental or therapeutic factor that changes the relative survival or net growth of competing lineages. Clonal fitness is therefore context dependent and refers to the capacity of a lineage to proliferate, survive, disseminate, and leave descendants in a defined environment. In patient-derived omics data, fitness is usually inferred indirectly from longitudinal expansion, persistence during treatment, enrichment in metastases, or recurrence after a bottleneck. Expression signatures labelled invasive, stem-like, or exhausted are state markers rather than direct measures of fitness.

Historical contingency, also termed phylogenetic inertia, refers to the constraint that prior genomic, epigenetic, and treatment history places on accessible future states. Truncal mutations, copy-number architecture, whole-genome duplication, lineage-specific chromatin, etiologic background, and previous therapy may render some resistance routes more accessible than others. Prediction should therefore consider both the current state and the evolutionary path by which it arose (9, 21, 22). **Table 1** summarizes the operational definitions, inference criteria, and principal caveats.

**Table 1:** Core evolutionary terms, operational definitions, and principal caveats.

Term	Operational meaning	How it is inferred	Principal caveat
Truncal alteration	Alteration assigned to the common ancestral lineage of the sampled tumor population.	Purity-, ploidy-, and copy-number-adjusted cancer-cell fraction; concordance across regions; or direct single-cell co-occurrence.	Limited sampling or low purity may cause a subclone to appear truncal or remain undetected.
Subclonal alteration	Alteration confined to a fraction of cancer cells or to one or more descendant lineages.	Cancer-cell fraction below 1.0, regional restriction, clustering of co-varying variants, or single-cell genotype.	Detection depends on sequencing depth, purity, copy number, and sampling density.
Branch	Alterations shared by one descendant lineage but absent from other sampled lineages.	Phylogenetic clustering across regions or cells.	Tree topology is model dependent and may not be unique.
Bottleneck	Reduction in effective population size or clonal diversity after seeding or treatment.	Loss of lineages, reduced diversity, or re-expansion from a restricted lineage in longitudinal samples.	Sparse sampling can mimic a bottleneck.
Selection pressure	Environmental or therapeutic factor that alters relative lineage survival or reproduction.	Reproducible enrichment with lineage expansion, longitudinal change, or a response to perturbation.	Association alone does not establish a selective effect.
Context-dependent fitness	Net growth, survival, dissemination, and production of descendants in a defined environment.	Longitudinal expansion, persistence during treatment, metastatic enrichment, recurrence, or validated functional competition.	Expression signatures are indirect proxies rather than direct measurements.
Neutral-like evolution	Subclonal frequency pattern compatible with genetic drift under a specified model.	Allele-frequency distribution after purity and copy-number correction.	Model violations or spatially restricted selection may produce an apparently neutral pattern.
Adaptive evolution	Differential expansion attributable to a heritable feature under selection.	Longitudinal or spatial lineage expansion, recurrent evidence of selection, and, ideally, functional validation.	Clone age, sampling, and variation in mutation rate can confound inference.
Historical contingency	Prior genomic, epigenetic, and treatment history constrains accessible future states.	Event ordering, lineage-specific states, and reproducible resistance routes.	Usually inferred retrospectively and difficult to test prospectively.

### 2.2 Neutral-like and adaptive evolution

Neutral models predict that many passenger-bearing subclones accumulate without sustained differences in net fitness, yielding characteristic allele-frequency distributions under specified assumptions (23). Adaptive models instead attribute repeated lineage expansion to a heritable feature that confers a selective advantage. Quantitative methods can test deviations from neutral expectations and estimate subclonal selection, but the result depends on sequencing depth, copy-number correction, tumor purity, sampling time, and the assumed population model (20, 24, 25).

Neutral-like and adaptive evolution need not be mutually exclusive within a tumor. A lesion may show near-neutral diversification during one interval, strong ecological or treatment-induced selection during another, and punctuated copy-number change during a later phase. An apparently neutral bulk pattern does not exclude spatially restricted selection, state-dependent advantages, low-frequency drivers, or variable mutation rates. Conversely, a recurrent molecular state does not establish adaptive causality without evidence of lineage expansion or functional evidence (13, 19–25).

Accordingly, we use neutral-like when the observed frequency distribution is compatible with drift under the stated model, and adaptive when longitudinal, spatial, phylogenetic, or perturbational data support differential lineage expansion. This terminology acknowledges the limits of inference from cross-sectional data.

### 2.3 Multi-omics platforms and the special contribution of single-cell DNA sequencing

Bulk whole-genome sequencing, whole-exome sequencing, and targeted panels characterize driver mutations, structural variants, mutational signatures, copy-number architecture, and phylogenetic relationships. Multi-region sampling distinguishes shared from private events and can reveal geographic segregation. Bulk measurements, however, combine genetically distinct cells and may miss rare clones when tumor purity or sequencing coverage is low (12, 20–22, 26–29).

Single-cell DNA sequencing resolves genotype and copy number at the level of individual cells. In HCC, single-cell and single-variant studies have revealed extensive genetic diversity, heterogeneous evolutionary modes, both punctuated and gradual copy-number evolution, and complex relationships among multifocal lesions (13–19). Interpretation is limited by allelic dropout, amplification bias, sparse coverage, doublets, and copy-number segmentation errors, all of which can affect tree topology. The evidence is strongest when single-cell DNA data are integrated with matched bulk sequencing, pathology, spatial information, and longitudinal clinical data.

Epigenomic assays characterize DNA methylation, chromatin accessibility, enhancer activity, and three-dimensional chromatin organization (30–34). Single-cell RNA sequencing and spatial transcriptomic or proteomic methods define malignant and microenvironmental states and their spatial neighborhoods (35–40), whereas proteomics and phosphoproteomics assess pathway activity that may not be evident from DNA or RNA alone. Serial ctDNA, methylation, fragmentomic, and circulating-cell assays provide longitudinal measures of molecular burden and emerging resistance (41–43). These platforms address different aspects of tumor evolution; integration is warranted only when it provides reproducible information beyond a focused assay. **Table 2** summarizes their contributions, potential clinical uses, readiness, and limitations.

**Table 2:** Contributions and limitations of major platforms for evolutionary inference.

Platform	Primary evolutionary contribution	Potential clinical use	Readiness and key limitation
Multi-region WGS/WES or targeted DNA	Truncal and branch architecture; mutational signatures; structural variants; allele-specific copy number; seeding relationships.	Risk stratification, trial enrichment, and design of tumor-informed ctDNA assays.	Established methods; limited by sampling, purity, coverage, and model assumptions.
Single-cell DNA sequencing	Cell-level genotype/copy-number co-occurrence, rare clones, and punctuated or gradual evolution.	Mechanistic subgrouping and identification of resistance lineages.	Research use; limited by allelic dropout, amplification bias, sparse coverage, and cost.
Methylation and chromatin assays	Regulatory memory, cell state, enhancer activity, field defects, and tissue-of-origin signals.	Early detection, subtype refinement, and methylation-informed MRD.	Promising; signals may reflect inflammation, cell of origin, tumor purity, or reversible state.
Single-cell and spatial omics	Malignant and microenvironmental states, spatial neighborhoods, ecological gradients, and treatment-adapted programs.	State biomarkers, candidate niche identification, and response signatures.	Discovery to early validation; cell state does not establish lineage, and spatial proximity does not establish causality.
Proteomics/phosphoproteomics	Pathway activity and signaling not reliably captured by DNA or RNA.	Target prioritization and pathway-informed combinations.	Limited standardization for routine use; substantial tissue requirements and uncertain temporal stability.
Serial liquid biopsy	Longitudinal tumor burden, molecular recurrence, emerging variants, and methylation or fragmentomic changes.	Surveillance, MRD assessment, resistance monitoring, and intervention within clinical trials.	Analytically mature for selected applications; clinical utility and control of confounding remain disease specific.
Integrated AI/ML models	Integrates multiple data types and clinical variables into risk or decision models.	Multimodal risk estimation and parsimonious decision-support tools.	Requires external validation, calibration, interpretability, fairness assessment, and comparison with simpler models.

### 2.4 Assumptions and failure modes of evolutionary inference

Phylogenetic reconstruction assumes that variants are observed accurately, that identical events do not arise independently, that back mutation is negligible, and that the mutation process is sufficiently stable to permit event ordering. Gastrointestinal cancers may violate these assumptions through loss of heterozygosity, whole-genome duplication, chromosomal instability, convergent pathway evolution, treatment-associated mutagenesis, and nonuniform mutation rates. Branch lengths may also be distorted because clone size reflects both clone age and fitness (9, 12, 20–22). Phylogenies should therefore be reported with uncertainty estimates and sensitivity analyses rather than presented as a single definitive history.

Tumor purity and sampling have similar effects on inference. Low purity reduces sensitivity for low-frequency variants and may cause a subclone to be classified as absent. A small number of biopsies sample only part of the tumor’s spatial diversity, while necrosis, mucin, stromal admixture, and treatment effects vary across regions. Multi-region sampling improves resolution but cannot exclude unsampled lineages. In multifocal HCC, intrahepatic metastases must also be distinguished from independent primary tumors.

Distinguishing drivers from passengers requires convergent evidence from recurrence above the expected background rate, functional annotation, clonality, pathway convergence, evolutionary timing, and experimental validation; algorithms may disagree (10, 25, 44). Copy-number inference is particularly sensitive to purity, ploidy, subclonal admixture, segmentation, and whole-genome duplication. RNA- derived copy-number estimates can identify broad malignant compartments but are not equivalent to allele-specific DNA copy number.

**Figure 1:**
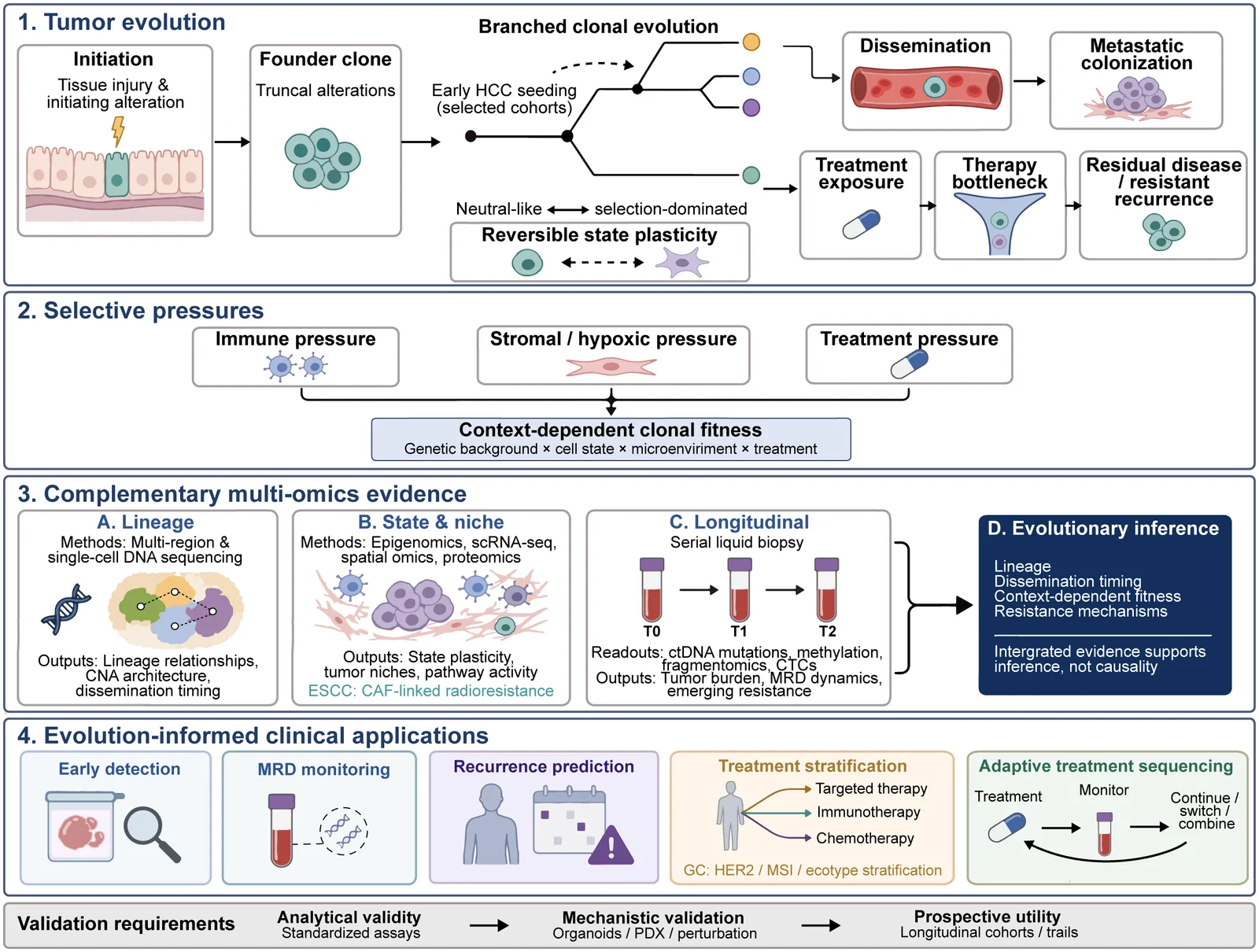
Multi-omics framework linking gastrointestinal cancer evolution to clinical translation. Tissue injury and initiating alterations give rise to a founder clone, followed by branched diversification that may range from neutral-like to selection-dominated dynamics. Reversible cell-state plasticity is shown separately from genetic lineage branching. Dissemination may arise from early or later lineages; the dashed route denotes early seeding reported in selected HCC cohorts. Treatment imposes a bottleneck that may leave residual or resistant clones, while immune, stromal, hypoxic, and treatment pressures shape context-dependent fitness. Multi-region and single-cell DNA sequencing resolve lineage architecture; epigenomic, single-cell RNA, spatial, and proteomic assays define cell states and niches; and serial liquid biopsy measures tumor burden, MRD, and emerging resistance. These data support inferences about lineage, dissemination, fitness, and resistance but do not by themselves establish causality. The lower modules summarize potential clinical applications and the analytical, mechanistic, and prospective validation required for implementation. Cancer-specific examples include early HCC seeding, ESCC CAF-associated radioresistance, and GC HER2/MSI/ecotype stratification. CAF, cancer-associated fibroblast; ctDNA, circulating tumor DNA; ESCC, esophageal squamous cell carcinoma; GC, gastric cancer; HCC, hepatocellular carcinoma; MRD, minimal residual disease; MSI, microsatellite instability; PDX, patient-derived xenograft.

Multi-omics integration is affected by batch effects, unequal feature scales, missing modalities, cell-composition confounding, and model instability. Correlations across layers may arise from a shared upstream process rather than a causal relationship. Cross-sectional tumor atlases sample limited tissue at a single time point, are vulnerable to dissociation and preservation bias, and are better suited to hypothesis generation than to establishing temporal sequence. **Figure 1** links complementary lineage, state, spatial, and longitudinal evidence to evolutionary inference and to the validation steps required for clinical use (12, 37–40, 45–47).

## 3 Genomic diversification and metastatic dissemination

### 3.1 Hepatocellular carcinoma

HCC evolves in a chronically injured organ in which viral hepatitis, alcohol-related disease, metabolic dysfunction, cirrhosis, and fibrosis shape the tissue background in which clones arise and are selected. Recurrent alterations in *TP53*, *CTNNB1*, *AXIN1*, telomere-maintenance pathways, chromosomal instability, and broad copy-number changes define major evolutionary routes (48–54). *TP53*-altered and copy-number-high tumors are frequently associated with aggressive behavior, whereas Wnt/beta-catenin activation is associated with immune exclusion and altered treatment responsiveness (48–53).

Single-cell DNA studies refine this genomic framework. Ling et al. described extreme cell-to-cell diversity consistent with extensive non-Darwinian diversification in one HCC (13), and Xue et al. showed that the degree and pattern of heterogeneity vary across patients and lesions (14). Duan et al. identified several evolutionary modes in HBV-related HCC (16), whereas Guo et al. reported both punctuated and gradual phases of copy-number evolution (19). Regional genomic-immune integration by Losic et al. and single-variant reconstruction by Su et al. further illustrated the complexity of HCC clonal histories (17, 18). Collectively, these studies do not support a single model of HCC clonal architecture.

Evidence for early dissemination is strongest in selected HCC cohorts. Multi-region and paired primary-metastasis analyses indicate that metastatic or intrahepatic lineages may diverge before extensive expansion of the clinically dominant primary tumor (15, 54, 55). Potential contributing features include vascular access, hypoxia, fibrosis, and early acquisition of traits that permit dissemination. Study design also influences this impression because HCC cohorts more often include multifocal lesions, broader liver sampling, and etiologically stratified. Early seeding should therefore be regarded as a recurrent, not universal, route, and recurrence studies must distinguish intrahepatic metastasis from a new primary arising in the diseased liver.

### 3.2 Esophageal squamous cell carcinoma

ESCC commonly develops within a chronically injured squamous field. *TP53* alterations, NOTCH pathway disruption, copy-number remodeling, and epigenetic drift can be detected before invasive cancer and provide a substrate for branched progression (56–64). Multi-region analyses have identified early tumor-suppressor events, later regional drivers, and substantial spatial heterogeneity in genomic and methylation profiles (61, 62). Whole-genome sequencing of large Chinese cohorts has further defined molecular features associated with poor prognosis (64).

The evolutionary setting differs from that of HCC. Selection operates across an extended epithelial field rather than within a cirrhotic parenchymal organ, and progression involves competition among premalignant clones, invasion across tissue boundaries, lymphatic dissemination, and treatment-associated selection. Studies including normal mucosa, primary tumors, and metastases support branched trajectories, but longitudinal paired metastatic cohorts remain limited (63). The timing of dissemination in ESCC is therefore less well resolved, owing both to disease biology and to the available study designs.

Stem-like and epithelial-mesenchymal-transition (EMT)-like programs may accompany progression, but transcriptional states should not be equated with distinct clones. Integration of lineage-resolved DNA, cell-state, and spatial data is needed to determine whether a resistant phenotype reflects expansion of a pre-existing genetic lineage, reversible adaptation across multiple lineages, or both.

### 3.3 Gastric cancer

GC comprises chromosomal instability, microsatellite instability, Epstein-Barr virus-associated, and genomically stable routes, together with HER2-amplified and clinically relevant transcriptional subgroups (65–70). *ARID1A* and *TP53* alterations, *ERBB2* amplification, mutation burden, and epigenetic programs interact with histology and immune-stromal context. Consequently, the implications of a given genomic alteration may differ among immune-active, immune-suppressed, and stroma-rich ecosystems.

Paired primary-metastasis studies do not support a single dissemination pattern for GC. Multi-region sequencing of advanced disease has identified both monoclonal and polyclonal seeding, and spatial profiling suggests that deeper invasive regions may be more closely related to nodal metastases than superficial regions (71, 72). These findings argue against a universal early-versus-late model. Dissemination appears to vary with subtype, depth of invasion, anatomic route, treatment, and the regions sampled.

Large-scale Asian sequencing studies and gastric cancer organoid resources extend the evidence base beyond TCGA and provide models for functional testing (70, 73). Genomic classification alone, however, is insufficient: intratumoral variation in HER2, PD-L1, MMR status, and stromal ecotypes may alter the predictive meaning of a single biopsy.

## 4 Epigenetic, transcriptional and metabolic plasticity

Non-genetic plasticity allows cancer cells to enter heritable or reversible states without acquiring a new canonical driver mutation. DNA methylation, chromatin accessibility, enhancer rewiring, three-dimensional genome organization, transcription-factor activity, RNA editing, and metabolic adaptation may change the fitness of a lineage. These states may be coupled to a particular genotype, induced across several lineages by a shared environment, or maintained only while a specific niche or therapy is present (30–34).

In HCC, chronic inflammation and fibrosis are associated with changes in DNA methylation and with Wnt, hypoxia, angiogenic, and metabolic programs (74–80). Etiology-linked epigenetic memory may influence which lineages tolerate oxidative stress, vascular deprivation, or immune pressure. Methylation-based ctDNA assays are attractive because they can combine tissue-of-origin information with tumor burden; associations with recurrence, however, require prospective defined thresholds and controls for background liver disease.

In ESCC, *SOX2*-related regulatory programs, *ADAR1* dependence, stemness, EMT-like transitions, and epithelial-stromal signaling may contribute to progression or treatment tolerance (59, 60, 81). These findings support plasticity as an evolutionary substrate, but pseudotime and expression similarity do not provide a lineage history. Paired measurements of genotype and cell state, together with perturbational studies, are needed to determine whether a state precedes, follows, or drives clonal expansion.

In GC, EBV-associated epigenetic remodeling, MSI-associated immune recognition, chromosomal instability, and transcriptional subtypes define distinct evolutionary contexts (65, 68, 69). Reversible states may alter HER2 expression, antigen presentation, metabolism, and stromal interaction. Mutation-based profiling alone may therefore miss clinically relevant resistance, whereas indiscriminate multi-omics profiling may add noise. The relevant clinical question is whether a limited set of complementary measurements improves a prespecified decision.

## 5 Microenvironmental selection: immune pressure, fibroblasts, hypoxia, and spatial niches

The tumor microenvironment imposes selective pressures through immune recognition, fibrosis, extracellular-matrix remodeling, hypoxia, angiogenesis, nutrient availability, microbiota-associated inflammation, and treatment-related tissue injury. These factors can alter the relative survival of competing lineages. Spatial and single-cell assays reveal associations between malignant and non-malignant compartments, but spatial proximity, ligand-receptor expression, and correlated transcription do not establish functional dependence (36–40, 46, 82–102).

In HCC, malignant hepatocyte-like populations coexist with exhausted or excluded lymphocytes, macrophages, fibroblasts, endothelial cells, and angiogenic niches (82–90). Hypoxia may favor invasive, metabolically adapted, and immune-evasive phenotypes (87). Fibrotic architecture can protect some regions while constraining others, creating spatially heterogeneous selection. These observations nominate candidate mechanisms but require co-culture, organoid, xenograft, lineage-tracing, or perturbation evidence before a niche can be considered causally selective.

In ESCC, reciprocal signaling between epithelial cells and fibroblasts is particularly prominent. Tumor or premalignant epithelial cells can activate fibroblasts, while fibroblast-derived extracellular matrix and cytokine programs may promote invasion and radioresistance (91–97). Collagen-associated *CXCL1* signaling and CAF-related signatures provide plausible mechanistic links, but prognostic associations do not establish therapeutic targets. Causal relevance requires evidence that disrupting these interactions alters lineage survival in experimental models and improves outcomes in an appropriately selected clinical population.

In GC, immune and stromal ecotypes, tertiary lymphoid structures, macrophage states, and fibroblast barriers may influence response to immunotherapy or targeted treatment (98–102). The relevant biological unit may be a spatial neighborhood rather than an individual cell type. Spatial tissue markers could complement routine pathology and plasma monitoring, but reproducibility across sampling sites, platforms, and treatment contexts is required.

## 6 Comparative evolutionary features of HCC, ESCC, and GC

HCC, ESCC, and GC share branched clonal architecture, non-genetic plasticity, context-dependent fitness, and treatment-induced selection, but the tissue context in which selection operates differs. HCC develops in a chronically diseased organ characterized by fibrosis, vascular remodeling, and the possibility of independent primary tumors. ESCC evolves across an injured epithelial field in which premalignant clones and stromal crosstalk influence invasion. GC includes multiple molecular and histologic routes with pronounced subtype- and ecotype-dependent behavior.

Fibrosis in HCC and field cancerization in ESCC both involve chronic inflammation, stromal remodeling, and epigenetic reprogramming, but their spatial effects differ. In HCC, fibrosis reorganizes perfusion, oxygenation, and immune access throughout a three-dimensional parenchymal organ. In ESCC, field cancerization produces genetically altered epithelial patches, increasing the opportunity for multiple precursor clones to progress. In GC, selection varies across glandular architecture and molecular subtypes, and immune-stromal ecotypes can alter the clinical significance of the same driver alteration.

The stronger evidence for early dissemination in HCC probably reflects both biological and study design. Vascular anatomy and hypoxic selection may facilitate early escape, while HCC cohorts also more often include multifocal lesions and broader sampling of the surrounding liver. Densely sampled longitudinal primary-metastasis datasets are less common in ESCC and GC, and the predominant routes of spread differ. Comparative conclusions should therefore distinguish limited evidence from evidence of a genuinely different trajectory (15, 54, 55, 61–63, 71, 72).

**Figure 2:**
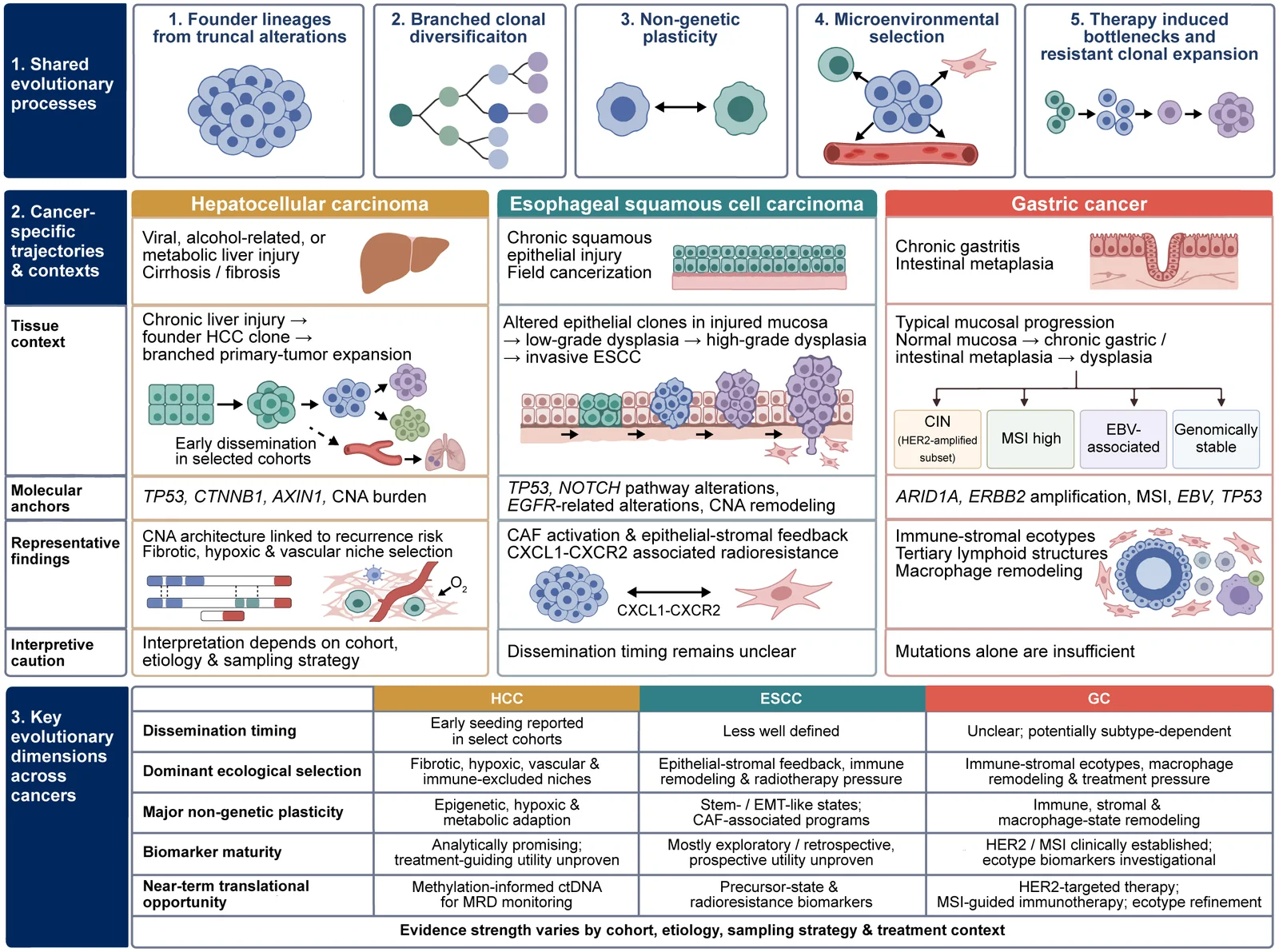
Comparative evolutionary trajectories and translational opportunities in HCC, ESCC, and GC. The upper band summarizes shared evolutionary processes and is not intended as a fixed temporal sequence. HCC arises in a chronically injured, fibrotic liver; early dissemination has been reported in selected cohorts; and copy-number architecture and hypoxic or vascular niches are associated with recurrence risk. ESCC evolves through field cancerization and stepwise dysplasia, with epithelial–CAF feedback and *CXCL1*–*CXCR2*-associated radioresistance. The GC panel shows a representative, non-universal mucosal progression route followed by divergence into CIN, HER2-amplified, MSI-high, EBV-associated, and genomically stable disease; immune–stromal ecotypes, tertiary lymphoid structures, and macrophage remodeling modify biomarker interpretation. The lower matrix compares dissemination timing, ecological selection, non-genetic plasticity, biomarker maturity, and near-term translational opportunities. Evidence strength depends on cohort, etiology, sampling strategy, and treatment context. CAF, cancer-associated fibroblast; CIN, chromosomal instability; CNA, copy-number alteration; ctDNA, circulating tumor DNA; EBV, Epstein–Barr virus; EMT, epithelial–mesenchymal transition; ESCC, esophageal squamous cell carcinoma; GC, gastric cancer; HCC, hepatocellular carcinoma; HER2, human epidermal growth factor receptor 2; MRD, minimal residual disease; MSI, microsatellite instability.

**Figure 2** and **Table 3** compare dissemination timing, selection pressures, non-genetic plasticity, biomarker maturity, near-term opportunities, and cancer-specific caveats across HCC, ESCC, and GC. Established tissue biomarkers remain the clinical reference standard. Longitudinal ctDNA and methylation assays require disease-specific prospective utility studies, whereas single-cell DNA and spatial fitness signatures remain primarily research tools for mechanistic studies and trial-enrichment.

**Table 3:** Comparative evolutionary dimensions in HCC, ESCC, and GC.

Dimension	HCC	ESCC	GC
Evolutionary substrate	*TP53*, *CTNNB1*, *AXIN1*, telomere maintenance, CNA burden, and etiology-linked methylation.	*TP53*/NOTCH disruption, CNA remodeling, field defects, and stem-like or EMT-like states.	CIN, MSI, EBV-associated epigenetic programs, genomically stable disease, HER2, *ARID1A*, *TP53*, and transcriptional subtypes.
Dissemination timing	Early or preclinical seeding supported in selected cohorts; estimates vary with etiology and sampling.	Branched progression and metastasis are supported; timing remains less well resolved because longitudinal paired cohorts are limited.	Monoclonal and polyclonal routes coexist; timing varies with subtype, depth, site, and treatment.
Dominant selection	Cirrhosis, inflammation, fibrosis, hypoxia, angiogenesis, immune exclusion, and treatment pressure.	Chronic epithelial injury, field competition, epithelial-CAF crosstalk, radiotherapy, and immune pressure.	Immune-active and stroma-rich ecotypes, macrophage-fibroblast crosstalk, targeted-therapy pressure, and immunotherapy.
Non-genetic plasticity	Methylation, Wnt/hypoxia programs, metabolism, and adaptation to vascular niches.	*SOX2*/*ADAR1* regulation, stemness, EMT-like transitions, and CAF-conditioned radioresistance.	EBV/MSI-associated programs, reversible transcriptional states, HER2/antigen variability, and stromal remodeling.
Biomarker maturity	Routine pathology and selected genomic assays; ctDNA/methylation MRD is promising but lacks universally validated action thresholds.	Routine pathology and established clinical predictors; postoperative ctDNA is promising, whereas spatial precursor and radioresistance markers remain experimental.	HER2, MSI/MMR, and PD-L1 are used clinically; ctDNA and ecotype-based refinement are promising, whereas spatial niche signatures remain experimental.
Near-term opportunity	Prospective validation of postoperative surveillance and methylation-informed MRD.	Longitudinal neoadjuvant sampling, tumor-informed MRD studies, and validation of radioresistance markers.	Prospective studies combining validated tissue biomarkers with serial ctDNA.
Specific caution	Multifocal lesions may represent metastases or independent primaries; background liver disease complicates the specificity of plasma and methylation signals.	Field defects blur the boundary between precursor and malignant tissue; metastatic sampling remains uneven.	Subtype mixing and spatially segregated ecotypes may make a single biopsy unrepresentative.

## 7 Therapy as an evolutionary selection pressure

### 7.1 Primary and acquired resistance and modality-specific bottlenecks

Primary resistance reflects a pretreatment tumor that lacks the relevant therapeutic vulnerability or is protected by a dominant cell state or microenvironmental feature. Acquired resistance develops after an initial response through expansion of a rare pre-existing lineage, acquisition of a new alteration, reversible adaptation, or microenvironmental protection. Baseline multi-region or single-cell sampling is most informative for primary resistance, whereas serial tissue or liquid-biopsy sampling is needed to establish acquired resistance.

Therapeutic modalities impose distinct but overlapping selective pressures. Targeted therapy may enrich on-target resistance mutations, bypass signaling, lineage switching, or loss of the targeted feature. Immunotherapy may select for antigen loss, impaired antigen presentation, interferon-pathway alterations, T-cell exclusion, or immunosuppressive niches. Radiotherapy may enrich cells or states with enhanced DNA repair, tolerance of hypoxia, favorable cell-cycle state, or fibroblast-mediated protection. Cytotoxic chemotherapy may select for drug efflux, enhanced repair, apoptotic tolerance, metabolic adaptation, or slow-cycling persister states. Each modality can create a bottleneck that reduces genetic diversity while enriching phenotypically plastic survivors.

In HCC, Wnt/beta-catenin-associated immune exclusion, angiogenic niches, and underlying liver disease may influence immunotherapy response (52, 53, 82–90). In ESCC, radiotherapy and neoadjuvant immunochemotherapy remodel both malignant and stromal compartments (91–97). In GC, HER2 heterogeneity, MSI/EBV status, immune ecotype, and stromal architecture may shape responses to targeted therapy and immunotherapy (65–69, 98–102). Longitudinal sampling is required to distinguish baseline predictive features from treatment-induced states.

### 7.2 Should subclones be targeted?

Targeting a non-dominant subclone may be reasonable when it is a credible precursor of relapse, but several uncertainties remain. A low-frequency event may be a passenger, may not be druggable at tolerable exposure, or may not persist. Eliminating one lineage can also release competing or cross-resistant lineages from ecological suppression. Conversely, failure to address a repeatedly expanding, mechanistically validated subclone may allow clinically relevant resistance to emerge.

Current practice should prioritize truncal dependencies and clinically validated dominant drivers. Subclonal targeting should be tested only when lineage identity, temporal expansion, targetability, and an action threshold are prespecified. Combination or sequential strategies require prospective evaluation rather than inferred from a single post-treatment sample. Evolutionary monitoring is most useful when it changes a defined clinical decision and the available interventions have distinct mechanisms.

### 7.3 Adaptive therapy, treatment sequencing, and evolutionary steering

Adaptive therapy modulates treatment intensity to preserve competition between sensitive and resistant cells rather than maximizing short-term cytoreduction in every cycle (103, 104). Evolutionary steering uses one treatment to shift the tumor toward a state that is more susceptible to a subsequent therapy (105). Clinical evidence for either strategy in HCC, ESCC, and GC remains limited, and both should be considered investigational.

An adaptive protocol requires a measurable population signal, a validated relationship between that signal and viable tumor burden, prespecified thresholds for escalation or de-escalation, a rapid assay, and therapies with sufficiently distinct resistance mechanisms. Potential triggers include a sustained rise in ctDNA, emergence of a validated resistance alteration, radiographic growth, or a combined molecular-clinical rule. Treatment changes should not be based on an isolated low-level variant because assay error, clonal hematopoiesis, and uncertain lineage fitness may confound interpretation.

Sequential strategies should be evaluated in trials that compare fixed and evolution-informed schedules, specify the proposed ecological mechanism, and assess patient-centered outcomes. Until such evidence is available, evolutionary data are best used for hypothesis generation, trial enrollment, and monitoring rather than off-protocol treatment switching.

## 8 Translational implementation

### 8.1 Liquid biopsy and minimal residual disease: prognostic validity does not establish clinical utility

Liquid biopsy permits serial sampling and can identify molecular recurrence before imaging in selected settings (41–43, 78–80, 106–109). Its evaluation requires three distinct levels of evidence. Analytical validity concerns whether the assay measures the intended signal reproducibly; clinical validity concerns whether the signal predicts recurrence or response; and clinical utility concerns whether acting on the result improves outcomes relative to standard care (110, 111). A strongly prognostic assay does not necessarily have treatment-guiding utility.

Colorectal cancer studies illustrate why prognostic performance and clinical utility must be distinguished. In stage II colon cancer, DYNAMIC reduced adjuvant chemotherapy use without compromising recurrence-free survival, with durable follow-up (112, 113). CIRCULATE-Japan studies have shown strong prognostic stratification and associations between molecular residual disease and benefit from adjuvant therapy (114, 115). DYNAMIC-III did not demonstrate uniform benefit across escalation and de-escalation strategies, and TRACC is prospectively testing ctDNA-guided treatment allocation (116, 117). These findings are informative but cannot be extrapolated directly to HCC, ESCC, or GC.

For HCC, ESCC, and GC, postoperative ctDNA or methylation positivity should currently be interpreted as a high-risk signal with evolving treatment-guiding utility. Trials should prespecify the sampling window, assay, threshold, confirmatory rule, intervention, comparator, and endpoint. A negative result does not exclude residual disease in low-shedding tumors, and earlier molecular detection does not by itself show that earlier treatment improves survival.

### 8.2 Clonal hematopoiesis and other liquid-biopsy confounders

Clonal hematopoiesis (CH) can contribute plasma cell-free DNA variants that are unrelated to the tumor, including alterations in genes also mutated in solid cancers. This is particularly consequential in early detection and MRD, where tumor fractions are low and a false-positive result may prompt unnecessary investigation or treatment. CH can also confound resistance monitoring when a persistent blood-cell clone is interpreted as tumor evolution (118–120).

Mitigation strategies include paired white-blood-cell sequencing when feasible, tumor-informed assay design, gene- and context-aware filtering, orthogonal confirmation, and assessment of serial kinetics. Very low variant allele fractions require cautious interpretation. A plasma variant detected in leukocytes, absent from tumor tissue, and stable despite tumor response should not be considered tumor derived. Methylation and fragmentomic assays reduce reliance on individual variants but introduce their own tissue-of-origin and inflammatory confounders.

Pre-analytical factors also affect sensitivity, including collection tube, processing delay, plasma volume, DNA extraction, sequencing depth, error suppression, and reporting thresholds. Reports should state the limit of detection, whether leukocyte DNA was analyzed, whether the assay is tumor informed, and what action threshold, if any, has been clinically validated.

### 8.3 Multi-omics versus focused assays and the role of artificial intelligence

Clinical use of multi-omics depends on demonstrable incremental value over focused testing. For some treatment decisions, a targeted DNA panel combined with immunohistochemistry may be sufficient. Single-cell or spatial profiling is better reserved for questions that cannot be resolved with standard assays, such as defining a mechanism, identifying a trial population, or improving prediction beyond established clinical variables.

A tiered framework is therefore more practical. Tier 1 consists of routine pathology, validated immunohistochemistry, focused genomic testing, and serial ctDNA in settings with established analytical and clinical validity. Tier 2 comprises reflex-expanded genomic, transcriptomic, methylation, or proteomic profiling for unresolved high-risk cases and prospective studies. Tier 3 includes discovery-grade single-cell and spatial assays. Movement between tiers should require evidence of incremental discrimination, calibration, decision impact, cost-effectiveness, and external validation.

Artificial intelligence and machine learning can integrate heterogeneous data, estimate risk, and derive parsimonious decision-support models (121, 122). Important limitations include data leakage, overfitting, site-specific batch effects, missing-data bias, poor calibration, and limited interpretability. Models should be evaluated in external and temporally separated cohorts, compared with simpler alternatives, and audited across demographic and clinical subgroups. Fairness assessment is essential because unequal access, limited ancestry representation, and differences in care pathways can translate technical bias into health inequity (123).

### 8.4 Logistical, economic, ethical, and regulatory requirements

Routine evolutionary monitoring depends on more than assay accuracy. Cost, reimbursement, sample logistics, tissue consumption, turnaround time, laboratory accreditation, regulatory approval, data storage, interoperability, and clinician education all determine whether a test can influence care. Even an accurate result has limited utility if it is returned after the treatment decision or requires expertise unavailable in routine practice.

Ethical considerations include consent for serial genomic testing, incidental germline findings, re-identification risk, subclonal findings of uncertain significance, the psychological effect of molecular relapse without an effective intervention, and equitable access. Patients should be informed whether a positive result is prognostic only or linked to an evidence-based action. Governance should specify responsibility for reviewing complex results, communicating uncertainty, and revalidating models after updates.

Health-economic evaluation should compare a tiered strategy with standard care and include downstream biopsies, imaging, treatment, false-positive results, and missed disease. Implementation should favor assays that are reproducible across laboratories and populations rather than those that simply maximize data generation. **Table 4** summarizes the readiness of major applications and the evidence required before routine adoption.

**Table 4:** Translational applications ranked by readiness and evidentiary requirements.

Application	Minimum practical assay	Current readiness	Decision and evidence required
Established therapy selection	Validated pathology/IHC with focused DNA or RNA testing.	Routine for established disease-specific indications.	Use validated disease-specific standards; any added omics must show incremental clinical utility.
Post-treatment MRD surveillance	Standardized tumor-informed or validated plasma assay, with paired WBC control where relevant.	Strong prognostic validity; treatment-guiding utility remains under evaluation.	Prespecified sampling window, cutoff, confirmatory rule, intervention, comparator, and prospective action-linked endpoint.
Recurrence-risk refinement	Clinical variables combined with selected tissue, CNA, immune-stromal, or methylation features.	Promising; prospective validation required.	External calibration, a reproducible score, incremental value, and a defined surveillance or adjuvant decision.
Resistance monitoring	Serial ctDNA with imaging; tissue biopsy when feasible.	Feasible in trials and selected targeted-therapy settings.	A validated resistance alteration, reproducible kinetics, and an evidence-based alternative treatment.
Spatial or single-cell fitness biomarkers	Research-grade spatial, scDNA, or scRNA assay with orthogonal validation.	Experimental.	Analytical simplification, functional evidence of causality, multisite replication, and prospective trial enrichment.
Adaptive therapy/evolutionary steering	Rapid quantitative biomarker and a prespecified treatment algorithm.	Investigational in HCC, ESCC, and GC.	Randomized comparison with fixed treatment, mechanistic confirmation, and patient-centered benefit.
AI/ML multimodal decision support	Harmonized inputs, a locked model, and uncertainty reporting.	Early clinical validation.	External and temporal validation, calibration, fairness assessment, interpretability, workflow integration, and regulatory oversight.

## 9 Unresolved controversies and requirements for functional validation

Key unresolved questions include how often neutral-like and adaptive phases coexist within the same tumor, when a transcriptional state is stable enough to be treated as an evolutionary unit, and which spatial niches exert causal selection rather than merely colocalize with specific clones. It is also unclear when subclone-directed therapy prevents relapse or instead releases a more resistant competitor, whether broad multi-omics provides sufficient decision value to justify cost and complexity, and which MRD-triggered interventions improve survival in HCC, ESCC, or GC.

Human tumor atlases are indispensable for hypothesis generation but have structural limitations. Most are cross-sectional, sample only a small fraction of the lesion, underrepresent rapid temporal change, and are sensitive to dissociation, preservation, annotation, and platform effects (46, 47). Inferred cell-cell communication is a statistical association rather than evidence of signaling dependence, and pseudotime orders states by similarity rather than chronological ancestry.

Functional validation should be matched to the claim. Organoids and patient-derived xenografts can test cell-intrinsic dependencies, whereas spatially informed co-cultures can evaluate fibroblast, endothelial, or immune interactions; CRISPR or pharmacologic perturbation can assess causality, lineage barcoding and single-cell DNA can connect cell state with ancestry; and prospective cohorts can test temporal prediction (73, 91, 92, 97, 99). Because no model captures the full human ecosystem, convergent evidence from complementary systems is preferable.

Mechanistic evidence must then be linked to a clinical decision by specifying the target population, assay, cutoff, intervention, comparator, and endpoint. A credible translational pathway proceeds from atlas-based association to perturbational validation, assay development, external validation, and prospective utility testing rather than direct from correlation to treatment recommendation.

## 10 Priority roadmap

Near-term work should focus on standardizing tissue and plasma assays already linked to established clinical decisions, including HER2 and MSI/MMR testing in GC and validated genomic or pathological features in each cancer. Disease-specific MRD studies should use fixed sampling windows, paired leukocyte controls where relevant, and prespecified clinical actions. In selected high-risk cohorts, multi-region tissue sampling should be combined with serial plasma analysis to determine when relapse-driving lineages become detectable.

Medium-term studies should determine whether a limited combination of genomic, methylation, immune, and stromal measurements improves recurrence prediction or therapy selection beyond focused assays. Models should report calibration, uncertainty, failure modes, and performance across sites and populations. Spatial and single-cell platforms are best used to identify mechanisms and trial-enrichment markers until their analytical workflows can be simplified and standardized.

Longer-term goals include lineage-resolved monitoring, adaptive treatment trials, and evolutionary steering. These strategies require rapid assays, therapies with nonoverlapping resistance mechanisms, and evidence that treatment changes at a molecular threshold improve survival, quality of life, or other patient-centered outcomes. Evolution-directed intervention should follow, rather than precede, validation of focused biomarkers, MRD-guided utility, and multimodal risk models.

## 11 Conclusions

Clonal evolution in HCC, ESCC, and GC reflects the interaction of genomic diversification, copy-number change, epigenetic and transcriptional plasticity, and tissue-specific ecology. Single-cell DNA sequencing strengthens lineage reconstruction by resolving cell-level co-occurrence and revealing heterogeneous, gradual, and punctuated evolutionary patterns. Single-cell and spatial maps provide complementary information on cell state and microenvironment, but they do not establish lineage or causality without independent evidence.

Cross-cancer comparison is clinically relevant. Evidence for early dissemination is strongest in selected HCC cohorts, although interpretation is complicated by multifocality and underlying liver disease. ESCC is shaped by premalignant field selection and epithelial-stromal crosstalk, but longitudinal metastatic and treatment-response datasets remain limited. GC follows subtype- and ecotype-dependent routes in which validated biomarkers coexist with marked spatial heterogeneity. These contrasts reflect both disease biology and differences in sampling and study design.

Translation should follow an evidence hierarchy in which focused assays remain the clinical reference standard, multi-omics is adopted only when it adds decision value, and MRD assays are evaluated separately for analytical validity, prognostic performance, and treatment-guiding utility. Spatial biomarkers, adaptive therapy, and evolutionary steering need to be prospectively tested. Progress will depend on showing that a reproducible evolutionary measurement changes a decision and improves patient outcome.
